# Evaluation of Rapid Antigen Test as a Marker of SARS-CoV-2 Infectivity

**DOI:** 10.7759/cureus.36962

**Published:** 2023-03-31

**Authors:** Akshay K Arya, Atul Garg, Sourav Pal, Richa Sinha, Nidhi Tejan, Ankita Pandey, Ujjala Ghoshal

**Affiliations:** 1 Microbiology, Sanjay Gandhi Postgraduate Institute of Medical Sciences, Lucknow, IND; 2 Microbiology, Indira Gandhi Institute of Medical Sciences, Patna, IND

**Keywords:** sars-cov-2, healthcare workers, infectivity, rat, sub-genomic

## Abstract

Background

Coronavirus disease 2019 (COVID-19) caused by severe acute respiratory syndrome coronavirus 2 (SARS-CoV-2) that emerged in late 2019 continues to spread globally. Reverse transcriptase polymerase chain reaction (RT-PCR), which is considered the gold standard for diagnosis, does not always indicate contagiousness. This study was planned to evaluate the performance of the rapid antigen test (RAT) with the duration of symptoms and the usefulness of these tests in determining the infectivity of patients by performing sub-genomic RT-PCR.

Methodology

This prospective, observational study was designed to compare the diagnostic value of the COVID-19 RAT (SD Biosensor, Korea) with COVID-19 RT-PCR (Thermo Fisher, USA) by serial testing of patients. To evaluate the infectivity of the virus, sub-genomic RT-PCR was performed on previous RAT and RT-PCR-positive samples.

Results

Of 200 patients, 102 were positive on both RT-PCR and RAT, with 87 patients serially followed and tested. The sensitivity and specificity of RAT were 92.73% and 93.33%, respectively, in symptomatic patients. The mean duration of RAT positivity was 9.1 days, and the mean duration of RT-PCR positivity was 12.6 days. Sub-genomic RT-PCR test was performed on samples that were reported to be positive by RAT, and 73/87 (83.9%) patients were found to be positive. RAT was positive in symptomatic patients whose duration of illness was less than 10 days or those with a cycle threshold value below 32.

Conclusions

Thus, RAT can be used as the marker of infectivity of SARS-CoV-2 in symptomatic patients, especially in healthcare workers.

## Introduction

The novel coronavirus, severe acute respiratory syndrome coronavirus 2 (SARS-CoV-2), emerged in December 2019 in Wuhan, China [1]. Within a few months, it spread globally, threatening human life. The World Health Organization (WHO) declared it a pandemic on March 11, 2020 [2]. So far, India has faced three waves of the coronavirus disease 2019 (COVID-19) pandemic, becoming one of the worst-affected countries worldwide [3]. The infection rate during the first wave caused by the original Wuhan strain was 0.744% which escalated to 1.11% (11,102 per 10 lakh population) during the second wave [3]. The third wave caused by the SARS-CoV-2 Omicron variant had a high transmission rate and low pathogenicity, resulting in countless clinical COVID-19 cases that were neither reported to the hospital nor were they evaluated. Therefore, the data remain unclear [4].

For controlling the pandemic, it is essential to understand the pattern and the duration of infectivity of SARS-CoV-2 [5]. Highly sensitive and specific tests are crucial in accurately identifying and managing patients with COVID-19. The gold standard test for the detection of acute SARS-CoV-2 infection is reverse transcriptase polymerase chain reaction (RT-PCR) [6]. A positive RT-PCR test does not necessarily translate to infectivity as it just detects the presence of viral RNA in the sample. It fails to discriminate if the virus is viable and transmissible or non-viable. Viable virus or active viral replication of SARS-CoV-2 can be confirmed by viral culture [7]. It has been documented that in the absence of a virus culture facility, RT-PCR tests targeting viral sub-genomic mRNAs can be performed directly on clinical samples to identify viable SARS-CoV-2 based on the fact that sub-genomic mRNA is transcribed only in infected cells, therefore, indicating the presence of actively infected cells in the sample [7].

To specifically detect antigens of the SARS-CoV-2 virus, reliable, cost-effective, and faster diagnostic tests have been developed [7]. These rapid antigen tests (RATs) have been approved by the WHO and are designed to detect SARS-CoV-2 nucleocapsid proteins coded by the N gene in respiratory secretions. Several variants of concern have originated in the last year and are associated with several S gene mutations; thus, they can be missed on the RT-PCR test. As RAT is based on nucleocapsid (N) antigens, it is highly unlikely to miss variants [8].

During the peak of COVID-19 infections, a vast majority of healthcare workers (HCWs) were infected. To protect patients HCWs rejoined duties once they were RT-PCR negative, which likely took several weeks [9]. It was our institute’s policy that HCWs needed to be RT-PCR negative before rejoining duties during the second COVID-19 wave caused by the delta variant. Usually, infectious virus is not detected in respiratory tract samples taken after eight days of symptom onset despite continued detection of high levels of viral RNA, which may lead to positive RT-PCR [10]; hence, if we wait for RT-PCR-negative reports for joining, it may lead to loss of working days of HCWs. Currently, there are no commercially available kits to identify infective COVID-19 patients and HCWs. Viral culture and sub-genomic RT-PCR can be performed only at reference laboratories. Therefore, it is important to develop a test that can identify infective COVID-19 patients. Recently, some studies have linked RAT tests in relation to the infectivity of patients, and this data can help in the implementation of control measures for quarantine, isolation, and contact tracing of HCWs [11]. As HCWs continue to perform their duties despite a risk of infection, exhaustion, and fear of transmission of infection to family members, tests are needed to decrease their apprehension and mental stress, which, in turn, will benefit patients. Thus, this study aimed to evaluate the diagnostic performance of RATs with the duration of symptoms and the usefulness of RAT in determining the infectiveness of patients.

## Materials and methods

This prospective, observational study was designed and conducted at the Department of Microbiology, Sanjay Gandhi Postgraduate Institute (SGPGI), Lucknow, India, and was approved by the Institutional Ethics committee (2020-326-IMP-EXP-33). The study was conducted between April and August 2021. Consecutive patients and HCWs presenting at the Emergency Department, Rajdhani Corona Hospital (RCH), SGPGI, Lucknow were recruited. Inclusion criteria included patients with fever, malaise, cough, sore throat, myalgia, arthralgia, headache, coryza, diarrhea, anosmia, or ageusia who presented within 48 hours of symptoms. The combined naso and oropharyngeal swabs were collected in viral transport media (VTM) for RT-PCR. The same microbiology technical staff performed bedside RAT tests on nasopharyngeal swabs. VTM were transported at 2-8°C to the Microbiology Laboratory, SGPGI, Lucknow for further processing. All VTM were processed in biosafety level 2 facilities with full personal protective equipment (PPE). Patients who tested positive for both RT-PCR and RAT were included in the study. Repeat serial samples were collected from these patients on days three, six, nine, 12, and 15 for both RT-PCR and bedside RAT.

Rapid SARS-CoV-2 antigen detection assay

STANDARD™ Q COVID-19 Ag Test (SD Biosensor, Inc, South Korea) was used for antigen detection per the manufacturer’s recommendations [12]. This is a rapid lateral flow assay for the detection of SARS-CoV-2 nucleocapsid (N) antigen in respiratory specimens. It has two pre-coated lines on the result window, namely, control and test lines. The test region is coated with mouse monoclonal anti-SARS-CoV-2 antibody against SARS-CoV-2 nucleocapsid (N) antigen. The antigen-antibody color particle complex migrates via capillary force and is captured by the test (T) region, which is coated by the mouse monoclonal anti-SARS-CoV-2 antibody. Nasopharyngeal samples were applied on a test device, and the test results were read in 15-30 minutes. For a positive COVID-19 antigen result, two colored lines of control (C) and test (T) lines were detected.

SARS CoV-2 detection by RT-PCR

RNA Extraction

RNA extraction was done using 200 μL of VTM using MagMAX Viral/Pathogen Nucleic Acid Isolation Kit (Thermo Fisher Scientific, Massachusetts, USA) using Kingfisher flex (Thermo Scientific, Massachusetts, USA) bench-top, 96-well, automated extraction platform per manufacturer’s instructions.

RT-PCR for SARS CoV-2 Detection

For genomic SARS-CoV-2 detection, a 25 μL RT-PCR reaction was prepared for the detection of SARS-CoV-2 by utilizing 5 μL of extracted RNA. Applied Biosystems TaqPath COVID-19 RT-PCR Kit Assay which targets ORF-1ab, spike (S) protein, and nucleocapsid (N) protein regions of SARS-CoV-2 was used for SARS-CoV-2 RNA detection according to the manufacturer’s instructions [13]. The RT-PCR cycling was performed at 55°C for 10 minutes for reverse transcription, followed by 95°C for three minutes, followed by 40 cycles of 95°C for 15 seconds and 58°C for 30 seconds using Quant Studio 5 RT-PCR system (Thermo Fisher Scientific, Massachusetts, USA). A cycle threshold (Ct) of less than 37 for all three target genes was taken as the cut-off for the detection of SARS-CoV-2 RNA according to the manufacturer’s instructions.

For sub-genomic SARS-CoV-2 detection, RT-PCR was performed as described by Wölfel et al. [10]. The oligonucleotide sequence of the leader-specific primer was as follows: sgLeadSARSCoV2-F; CGATCTCTTGTAGATCTGTTCTC. The sgRNA RT-PCR assay used the Superscript III one-step RT-PCR system with Platinum Taq Polymerase (Invitrogen, Darmstadt, Germany) with 400 nM concentrations of each of the primers, as well as 200 nM of the probe. Thermal cycling involved 10 minutes at 50°C for reverse transcription, followed by three minutes at 95°C, 45 cycles of 10 seconds at 95°C, 15 seconds at 56°C, and five seconds at 72°C [10].

Statistical analysis

Sensitivity, specificity, positive predictive value (PPV), negative predictive value (NPV), diagnostic accuracy, and 95% confidence intervals (CIs) were calculated using SPSS software version 26.0 (IBM Corp., Armonk, NY, USA).

## Results

Comparison between RT-PCR and the RAT

RT-PCR and RAT were performed on consecutive 200 patients presenting at the Emergency Department, RCH, SGPGI, Lucknow. Overall, 110/200 (55%) were RT-PCR positive, and 108/200 (54%) were RAT positive. Considering RT-PCR as a reference test, the sensitivity, specificity, PPV, NPV, and diagnostic accuracy of RAT were 92.73%, 93.33%, 57.91%, 99.24%, and 93.28%, respectively (Tables 1, 2).

**Table 1:** Comparison of the positivity rates of RAT with RT-PCR results. RAT: rapid antigen test; RT-PCR: reverse transcriptase polymerase chain reaction

	RT-PCR positive	RT-PCR negative	Total
Antigen positive	102	6	108
Antigen negative	8	84	92
Total	110	90	200

**Table 2:** Statistical analysis of study data.

Statistic	Value	95% confidence interval
Sensitivity	92.73%	86.17% to 96.81%
Specificity	93.33%	86.05% to 97.51%
Positive predictive value	57.91%	38.80% to 74.91%
Negative predictive value	99.24%	98.52% to 99.61%
Diagnostic accuracy	93.28%	88.87% to 96.33%

Of the 102 patients positive on both RT-PCR and RAT, six patients died during the course of treatment, and nine patients were discharged on request. Hence, 87 patients were followed up and serially tested further for both RAT and RT-PCR. Serial samples were collected until they tested negative or until day 15 whichever was earlier.

Mean duration of positivity

A total of 352 samples for antigen testing (range: 2-5 samples per person) and 453 (range: 4-6 samples per person) samples for RT-PCR testing were collected from these patients.

**Figure 1:**
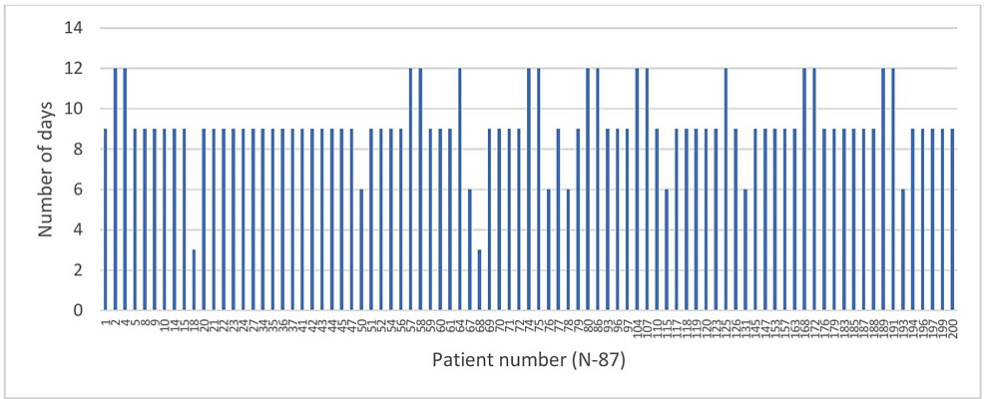
Positivity in days for rapid antigen test.

From symptom onset, 62/87 (71.2%) patients were RAT positive until nine days. The mean duration of antigen positivity was 9 ± 1.78 days. The longest duration for antigen positivity was found until 12 days (Figure 1).

**Figure 2:**
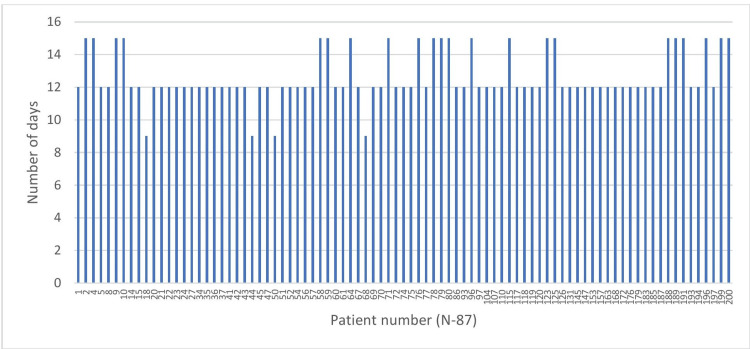
Positivity in days for reverse transcriptase polymerase chain reaction test.

A total of 61 (70.1%) patients were RT-PCR until 12 days from symptom onset. The mean duration of RT-PCR positivity was 12.6 ± 1.51 days. The longest duration for RT-PCR positivity was until 15 days (Figure 2).

The mean Ct value for RAT was found to be 30.4 ± 2.

Relationship of sub-genomic RT-PCR results with RAT

Sub-genomic RT-PCR results showed that 73/87 (83.9%) patients were positive. Of the 73 positive patients, the duration of illness was less than 10 days in 61/73 (83.5%) patients. In 12/73 (16.4%) patients, the duration of illness was more than 10 days (Table 3).

**Table 3:** Ct value of RAT-positive and sub-genomic RT-PCR-positive sample with duration of illness of more than 10 days. Ct: cycle threshold; RAT: rapid antigen test; RT-PCR: reverse transcriptase polymerase chain reaction

Patient number	Day until positive	Ct value
2	12	29
4	12	31
57	12	31
58	12	30
75	12	30
80	12	29
86	12	32
107	12	32
125	12	30
172	12	32
181	12	32
191	12	30

Of the 14 patients with negative sub-genomic RT-PCR, 10 patients had less than 10 days of duration of illness. The Ct value in sub-genomic RT-PCR was 32 or more. Duration of illness was higher in four patients (Table 4).

**Table 4:** Ct values of RAT-positive and sub-genomic RT-PCR-negative samples. Ct: cycle threshold; RAT: rapid antigen test; RT-PCR: reverse transcriptase polymerase chain reaction

Patient number	Days until positive	Ct value
1	9	32
21	9	33
34	9	34
44	9	35
52	9	33
56	9	31
97	9	33
18	9	32
120	9	33
199	9	33
64	12	33
74	12	32
104	12	32
168	12	34

Relation between RT-PCR and sub-genomic RT-PCR

Sub-genomic RT-PCR was performed on 87 VTM (the last sample of patients was collected between days nine and 12) that were RT-PCR positive. Results showed that sub-genomic RT-PCR was positive in six samples, and all positive patients had a duration of illness of fewer than 10 days. Of these, 3/6 patients had a Ct value of sub-genomic RT-PCR below 32. Among 81 patients whose sub-genomic RT-PCR was negative, 3/81 (3.7%) patients had a duration of illness of fewer than 10 days, and the Ct values of all three patients were above 32. The remaining 78 patients had a duration of illness of more than 10 days, and their RT-PCR Ct value was above 32.

## Discussion

During the COVID-19 pandemic, HCWs were at the heart of the crisis. While performing their duties with the utmost care, they were getting infected by the virus, leading to a more psychological burden, which was the case for COVID-19 patients as well. There is a need for a test to identify as many patients who are transmitting infection so that they can be isolated and their contacts can be identified and quarantined, which will also help in controlling the outbreaks. As we know RT-PCR can pick up dead viruses which can amplify and give positive RT-PCR results. Moreover, it does not indicate the infectiveness of the virus [14]. In our study, RAT-positive results coincided with the sub-genomic RT-PCR results, indicating active viral replication.

In our study, RAT showed a sensitivity of 92.73% and a specificity of 93.3% for symptomatic patients and HCWs. The diagnostic accuracy of the SARS-CoV-2 RAT test was 93.28% in detecting SARS-CoV-2 in N&P samples compared to RT-PCR as the gold standard test. A study by Gremmels et al. on the comparison of RAT and RT-PCR at two different sites in the Netherlands showed a specificity of 100% and sensitivity of 72.6% and 81.0% in the city of Utrecht (n = 1,367) and Aruba (n = 208) in the Netherlands, respectively. Taking Ct value <32 as a cut-off, sensitivity was 95.2% in Utrecht and 98.0% in Aruba. Overall sensitivity was 95.2% using Ct values <32 as the cut-off [15]. In another study done in Spain by Linares et al., a sensitivity of 86.5% was reported among individuals with symptoms for less than seven days [16]. The sensitivity of our test was comparable with these studies but the specificity was less in our study. In the study by Boum et al., symptomatic patients showed 94.0% sensitivity and 91.0% specificity in the first seven days after symptom onset for antigen detection test [17]. This study showed comparable results with our study regarding specificity. When comparing the RT-PCR as the standard test with the real-life clinical sensitivity, it showed that the sensitivity (95% CI) for laboratory-confirmed cases (repeat-tested patients) was 85.7% (81.5-89.1%); inpatients 95.5% (92.2-97.5%) and outpatients 89.9% (88.2-92.1%) [18]. These results demonstrate that RAT can be used as a promising alternative to RT-PCR. Dynamics of SARS-CoV-2 have demonstrated that viral replication and shedding are higher during the first few days of clinical disease and start decreasing eight to nine days after symptom onset [15,19].

The mean duration of antigen positivity was 9.1 ± 1.78 days (range: 3-12 days). In the study by Boumel et al. (n = 1,195), RAT positivity was 54.0%, during the first week of symptoms and then decreased. It also showed RAT positivity exceeded RT-PCR positivity on symptoms from days four to eight [17]. Another study also demonstrated higher diagnostic performance of RAT when used during the first five days following symptom onset due to the high viral load [20]. A meta-analysis by Xie et al. of 135 studies (n = 166,943 ) showed a decrease in the sensitivity of RAT as the days progressed. For symptom duration of ≤3, ≤7, and ≤10 days, the sensitivities were 0.91 (95% CI: 0.83-0.96), 0.89 (95% CI: 0.84-0.93), and 0.88 (95% CI: 0.83-0.92), respectively [21]. In our study, the RAT positivity decreased as the days progressed. It was expected as RAT does not amplify the virus present in clinical samples similar to what RT-PCR does. It can also be suggested that antigen positivity closely mirrors the phase of high viral shedding.

The mean duration of RT-PCR positivity was 12.6 ± 1.51 days. The longest duration for RT-PCR positivity was 15 days. In several other studies, the median duration from symptom onset to virus detection was 14.5 days in upper respiratory tract samples [22,23]. In lower respiratory tract samples, the median duration for virus detection from the onset of symptoms at the aggregated study level was 15.5 days [24,25]. The longest duration observed from the upper respiratory sample was 83 days in one patient [25]. Our results for RT-PCR positivity were comparable with other studies.

The high duration of RT-PCR positivity could be due to the amplification of non-viable virus particles, as reported by Walsh et al. that SARS-CoV-2 RNA can be detected for several weeks, long past when most people are infectious [26]. Viral culture denotes the viability and infectious nature of the virus. A viral culture study by Wolfel et al. found that no infectious isolates were cultured from the patient’s samples collected after day eight from symptom onset despite continued high viral load [10]. Another study by La Scola et al. also found that the virus could not be isolated from samples taken after day eight from symptom onset, although viral load remained high [27], indicating that RT-PCR positivity does not always denotes the infectious nature of the virus.

The mean (μ) Ct value for RAT positivity was 30.4 ± 2, whereas for RT-PCR positivity it was 35.3 ± 1.13. Ct values have been considered an arbitrary marker of infection. A study by Arons et al. showed that the lowest viral load (Ct value) for which there was positive culture growth was 34.3 [28]. Another study showed that there was no culture growth from samples with Ct values ≥34 targeting the E gene [27], suggesting that a Ct value below 34 can be taken as a marker of infectivity. Our study results for RAT positivity showed a mean Ct value of 30.4, which were consistent with the results of the viral culture studies which have accounted for Ct value, suggesting that RAT positivity can also be taken as a marker of infectivity, and the samples which are RAT negative should be considered as non-infectious.

Sub-genomic RT-PCR was also performed on the last positive sample of antigen and RT-PCR test of 87 patients. The study by Perera et al. showed that sub-genomic RNA and culturable viruses were not detectable after eight days from symptom onset in mild COVID-19 [29]. Research has also shown that sub-genomic RNA is a suitable marker for active infection as it degrades more rapidly than total RNA [30]. In our study, 73 antigen-positive samples were found to be positive for sub-genomic RT-PCR and 14 were negative for sub-genomic RT-PCR. Out of these 73 positive patients, 61 had a duration of illness of fewer than 10 days. As previously discussed, viral culture studies showed that no infectious isolates were obtained from any samples collected after day eight from symptom onset despite continuing to have a high viral load [18,27].

In our study, we found that the mean Ct for antigen positivity was 30.4 ± 2. Thus, it can be suggested that RAT positivity in symptomatic patients can be used as a marker of viral replication as antigen positivity was only found in patients whose duration of illness was fewer than 10 days or patients with Ct values below 32.

The limitation of the study was its small sample size. Furthermore, we could not perform RAT for different variants of SARS-CoV-2 as sequencing was not performed.

## Conclusions

The RAT showed excellent sensitivity and acceptable specificity for the N&P specimens in symptomatic patients. RAT positivity can be used as a marker of infectivity of SARS-CoV-2 in symptomatic patients and especially in HCWs as a negative result will decrease their psychological stress and increase their productivity. The assay is easy to use, cost-efficient, does not require expertise, and provides results within 30 minutes. Therefore, it has the potential to be a useful tool for early detection, a marker of infectivity for SARS-CoV-2, and in lowering the burden on laboratories and the government. However, in the case of asymptomatic patients, RAT should be used in conjunction with molecular tests, especially to rule out SARS-CoV-2 infection.
